# Cytogenetic and Biochemical Genetic Techniques for Personalized Drug Therapy in Europe

**DOI:** 10.3390/diagnostics11071169

**Published:** 2021-06-26

**Authors:** Tatjana Huebner, Catharina Scholl, Michael Steffens

**Affiliations:** Research Division, Federal Institute for Drugs and Medical Devices, 53175 Bonn, North Rhine-Westphalia, Germany; Catharina.Scholl@bfarm-research.de (C.S.); Michael.Steffens@bfarm-research.de (M.S.)

**Keywords:** companion diagnostic, personalized therapy, pharmacogenetics, pharmacogenomics, in vitro diagnostic, regulation

## Abstract

For many authorized drugs, accumulating scientific evidence supports testing for predictive biomarkers to apply personalized therapy and support preventive measures regarding adverse drug reactions and treatment failure. Here, we review cytogenetic and biochemical genetic testing methods that are available to guide therapy with drugs centrally approved in the European Union (EU). We identified several methods and combinations of techniques registered in the Genetic Testing Registry (GTR), which can be used to guide therapy with drugs for which pharmacogenomic-related information is provided in the European public assessment reports. Although this registry provides information on genetic tests offered worldwide, we identified limitations regarding standard techniques applied in clinical practice and the information on test validity rarely provided in the according sections.

## 1. Introduction

The European Medicines Agency (EMA) advises an inclusion of pharmacogenomic information in drug labels where an impact on particular genomic subpopulations is recognizable and provides guidance on the identification and validation of genomic biomarkers [1]. Thus, in recent years, the number of approved drugs with information on gene associated implications for therapy in the European public assessment reports (EPAR) is steadily rising [2]. A variety of genetic biomarkers for guided treatment and personalized therapy were identified and their utilization in companion diagnostics (CDx) is often supported by sufficient scientific evidence for preventive measures regarding adverse drug reactions and treatment failure [3,4]. In the European Union (EU), a companion diagnostic is defined by the new Regulation (EU) 2017/746 on in vitro diagnostic medical devices as a device that is of relevance “for the safe and effective use of a corresponding medicinal product” [5] to detect patients before and/or during use of the medicinal product who have a higher likelihood to benefit from the treatment or patients with a higher risk of serious adverse reactions due to the use of the medicinal product. The new Regulation also specifies that “the International Non-proprietary Name (INN) of the corresponding medicinal product” [5] shall be included in the instructions for use of a companion test [5].

For some drugs, genetic testing for relevant biomarkers is recommended or even mandatory prior to prescription. Rapid developments of genetic testing techniques and advancements in cost reduction for testing material and services are constantly increasing the amount of accessible and affordable genetic tests for clinical diagnostics on the market. Thus, many different protocols are available for the determination of the same biomarker. Therefore, the EMA does not provide guidance for the selection of a particular genetic test method as companion diagnostic. In the EPARs, generally the utilization of a validated test for the according biomarker is stated [3,4]. This approach shall promote the advancements in test development; however, for health care providers it complicates clinical decisions about the utilization of genetic tests for patient care. A comprehensive registry or data base that reflects the various types of available tests across the genetic testing landscape in Europe would therefore be crucial but is yet not available.

In the EU, the current Databank on Medical Devices (Eudamed2) is only used by national competent authorities and the European Commission with regard to market surveillance and is not publicly accessible. A new European database on medical devices (EUDAMED) will be established also to implement the new regulation (EU) 2017/746 on in vitro diagnostic medical devices, which will apply fully from 26. May 2022. The database is launched module wise starting by December 2020. EUDAMED will be in part accessible to the public and will increase transparency on medical devices available for the EU market including in vitro diagnostics like genetic tests [5,6].

In the United States of America (USA), an oversight of laboratories and offered genetic tests is provided by the Food and Drug Administration (FDA) and the Center for Medicare and Medicaid Services (CMS) respectively on federal level. Still, several regulatory gaps and ambiguities were reported [7]. To increase transparency, the National Institute of Health established the Genetic Testing Registry (GTR) providing a centralized, publicly accessible platform for the health care community with information on genetic tests offered worldwide. GTR defines a genetic test as an analysis used to identify heritable or somatic mutations, genotypes or phenotypes associated with disease and health. Thereby, the tested source can be human genes and chromosomes, deoxyribonucleic acid, ribonucleic acid and gene products. The provided data includes the test’s purpose, methodology, corresponding test validity and utility specifications. Furthermore, laboratory credentials and contacts are provided. However, the genetic test information is submitted voluntarily by the test provider [7]. Nevertheless, the database provides a sufficient overview of genetic testing methods currently offered for the application in clinical settings. Therefore, for this review the GTR database was used as a source to build an overview of relevant testing methods that are presently offered for the analysis of genetic biomarkers important for prescriptions of uniformly approved drugs in the EU. The focus is on methods applied to test for genes or pharmacogenomic biomarkers for which testing is recommended or mandatory according to EPARs of approved drugs in the EU. Additionally, tests for pharmacogenomic biomarkers were included if annotations on actionable pharmacogenomics are provided in an EPAR of the respective drug.

## 2. Materials and Methods

Drug label annotation categories “Testing required”, “Testing recommended” and “Actionable PGx (Actionable Pharmacogenomics)” provided by The Pharmacogenomics Knowledge Base (PharmGKB) were used to list EMA authorized drugs and related biomarkers for which diagnostic tests can be performed to personalize drug therapy (Supplementary Materials 1 and 2) [8]. A listing of drugs for which diagnostic testing is required or recommended prior to prescription encompassing drugs for the treatment of rare diseases and predominantly different cancers and a further list of drugs categorized as PharmGKB “Actionable PGx” with relevant pharmacogenomics information was compiled. The lists were crosschecked for up-to-datedness in August 2020 at the European Medicines Agency website providing the updated European public assessment reports and further information on currently authorized drugs uniformly approved for the European market. This updated overview encompassing drugs for which companion diagnostics could improve therapy was applied for a screening of the GTR database for genetic tests by relevant drugs and in a second approach by the corresponding biomarkers of interest. In some cases, a screening by biomarker resulted in findings of technique combinations which were not applied to test for the same markers and of inconclusive GTR information on whether all tests cover the biomarker of interest. Then additional information, if available, was gained from the respective provider websites. For EGFR e.g., the test NeoTYPE^®^ Discovery Profile for Solid Tumors encompasses a methodology of Immunohistochemistry, Fluorescence in situ hybridization and Next-Generation (NGS)/Massively parallel sequencing (MPS). However, according to the provider website only NGS is applied to test for EGFR mutations, which is not indicated and therefore misleading in the GTR entries [9]. Therefore, tests were excluded if the relevant biomarker annotated in the according EPAR was not analyzed via the offered methodology of interest for this review (Cytogenetics, Biochemical genetics) when screening by biomarker and also when screening by drug to receive the respective companion diagnostic tests for drug response. Tests were identified in the three test method categories “Cytogenetics”, Biochemical Genetics and “Molecular Genetics” distinguished by GTR with an emphasis on molecular genetic techniques. Here we discuss test methods of the category “Cytogenetics” and “Biochemical Genetics” that are more and more supplemented or replaced by molecular genetic techniques such as next generation sequencing. Molecular genetic techniques were only included if applied in a combined approach with cytogenetic and biochemical genetic techniques and will be assessed in detail in future analyses.

## 3. Results

### 3.1. Cytogenetic Techniques for Guided Drug Therapy

The screening of the GTR by drug resulted in only four drugs for which cytogenetic tests were registered covering the respective biomarker addressed in the according EPAR. For all of these drugs, testing is required in Europe. Fluorescence In Situ Hybridization was registered in the GTR as an offered method for all of these drugs. Furthermore, for Lapatinib, Trastuzumab and Trastuzumab emtansin Microarray analysis (e.g., Affymetrix GeneTitan^®^ MC, Illumina HiScan™SQ system) classified as “Molecular Genetics” and for Trastuzumab and Trastuzumab emtansin response additionally Bi-directional Sanger Sequence Analysis (e.g., via ABI 3130 XL or 3730) was registered in the GTR. For guided therapy with Crizotinib, also a combination of a cytogenetic and a molecular genetic technique was identified. Here, RT-PCR with gel analysis applied for chromosome breakage analysis was registered as a cytogenetic method to test for Crizotinib response in combination with RNA analysis by Allele-specific primer extension, a molecular genetic technique (Table 1).

A screening of the GTR by the relevant biomarker addressed in the respective EPAR of a drug resulted in a higher variety of offered tests and combinations of testing techniques registered. However, mostly cytogenetic tests were based on three cytogenetic techniques (Table 2) or a combination of these cytogenetic techniques e.g., with biochemical genetic techniques (e.g., immunohistchemistry, enzyme assays) or targeted approaches based on e.g., PCR, hybridization or sequencing are registered (Supplementary Material 1).

For about 55% of the biomarkers relevant for the listed 58 drugs uniformly approved in the EU for which genetic testing is either recommended or required according to PharmGKB, cytogenetic tests are registered in the GTR. The most frequent combinations were FISH with G-banding (10.3%) and FISH with NGS (10.3%). Furthermore, for chromosome breakage studies, RT-PCR with gel analysis was registered in combination with Allele-specific primer extension (ASPE) for 8.6% of the analyzed drug-biomarker pairs. Here, always anaplastic lymphoma kinase (ALK) was analyzed as genetic biomarker by this combined approach. Thereby, FISH was identified as a single technique approach for this biomarker. Apart from Cystic Fibrosis, which is a rare disease, all of these drugs for which cytogenetic tests can be used to guide therapy are applied in cancer treatment.

Although several cytogenetic methods were registered to test for biomarkers for which information on actionable pharmacogenomics are provided in an EPAR of the respective drug, these tests comprise a combined approach with several methods to test for a set of markers. However, the respective provider websites informed that different tests were used for certain markers and cytogenetic tests were not applied for the pharmacogenomic biomarkers of interest in the “Actionable PGx” listing. Therefore, no cytogenetic tests could be identified to test for the biomarkers relevant for the list of drugs for which annotations on actionable pharmacogenomics are provided in an EPAR of the respective drug (Supplementary Material 2).

#### 3.1.1. G-banding/Karyotyping

Giemsa (G)-banding is a cytogenetic method to visualize condensed chromosomes and to attain a visible karyotype using Giemsa stain. It is applied to identify and analyze individual chromosomes according to unique banding patterns for diagnostics of genetic diseases and cancers according to ploidy, chromosome abnormalities and rearrangements [11,13,14].

Skills and experience are crucial to analyze karyotypes which are frequently complex, especially if they are tumor related. This banding technique reliably gives the maximum level of about 550 bands per haplotype set (bphs) of band resolution for karyotyping [15]. However, imbalances that are undetectable by microscope analysis of G- banding patterns cannot be identified by this method. Here, fluorescence in situ hybridization (FISH) and comparative genomic hybridization (CGH) which combine a cytogenetic and DNA-based approach have an advantage over Giemsa-banding to identify small imbalances or such that are not distinguishable based on the analysis of banded chromosomes alone [16]. Thus, G-banding is highly informative but frequently not sufficient as a sole approach for the detection of complex genetic abnormalities [15]. However, due to other limitations of FISH and CGH for chromosomal analyses, they cannot provide a replacement for G-banding [16]. Chromosome banding analysis is still used as a standard to detect the t(9;22)(q34;q11) or BCR-ABL rearrangement, to monitor treatment response in e.g., chronic myeloid leukemia. However, confirmatory tests such as FISH or reverse transcription PCR (RT-PCR) also have to be applied [11,17].

#### 3.1.2. Fluorescence In Situ Hybridization (FISH)

Fluorescence in situ hybridization is a cytogenetic technique developed to detect target DNA of e.g., metaphase chromosomes or interphase nuclei and RNA with a high sensitivity and specificity in cells or tissues [18]. It can identify genetic disorders such as chromosomal abnormalities and even copy number variations (CNVs). A present or absent genetic alteration of interest can be analyzed directly by visualization of the hybridization signals by fluorescent microscopy. Especially in cancer treatment, the application of FISH as a diagnostic method to guide therapy with regard to genetic abnormalities is recommended or even determined in the summary of product characteristics for certain drugs as a confirmatory test method of choice if the IHC first-tier method results are equivocal (Table 1). Thus, FISH assays are applied in clinical diagnostics and can be used to guide targeted therapy [19,20].

Probe size and design, tissue type and laboratory validation affect sensitivity and specificity of this technique. Thus, it is labor-intensive, time-consuming, technically demanding and relatively costly. However, analytically valid test kits for the analysis of different clinically relevant conditions are commercially available and thus can be applied directly after tissue preparation [19]. Chromogenic In Situ Hybridization (CISH) and Silver In Situ Hybridization (SISH) can also be used to examine the chromosomal number, chromosomal translocations and gene copy number variations due to gene amplification. They differ from FISH only in labeling and detection. CISH Probes are labelled with haptens like biotin or digoxigenin, which are bound by peroxidase- or alkaline phosphatase-coupled reporter antibodies. Regions where those antibodies bind are visualized via an enzymatic color reaction using chromogenic substrates catalyzed by the reporter enzymes coupled to the antibodies [21,22].

For SISH, probes are labeled with dinitrophenol (DNP) and signal detection is performed adding an anti-DNP primary antibody. Furthermore, a secondary antibody conjugated to horseradish peroxidase, silver acetate, hydroquinone and hydrogen peroxide are added and initiate the reduction of silver ions to metallic silver in the nucleus, if the target sequence is present [23].

CISH and SISH show a similar sensitivity [24,25] and are less expensive alternative methods to FISH required to test for HER2 overexpression in guided therapy with Trastuzumab. For signal detection bright-field microscopy is sufficient. A further advantage of CISH and SISH is that the signal staining is permanent and does not diminish over time [21,22,23].

At present, FISH is the preferred cytogenetic method routinely applied e.g., for guiding targeted therapy to identify human epidermal growth factor receptor 2 (HER2) overexpression in breast cancer, human epidermal growth factor receptor 2 anaplastic lymphoma kinase (ALK) rearrangement in adenocarcinoma and BCR/ALB1 translocation in chronic myeloid leukemia [26] and can be applied to detect other gene rearrangements such as ROS proto-oncogene 1 receptor tyrosine kinase (ROS1) [27]. Furthermore, GTR registered FISH tests or combined methodology tests including FISH were identified for ERBB2 (HER2), EGFR (ErbB-1), CFTR, RET, TP53 and other biomarkers which are relevant to guide treatment with several EU authorized drugs (Supplementary Material 1).

#### 3.1.3. Comparative Genomic Hybridization

CGH was developed for the analysis of genomic alterations in cancer and allows the evaluation of genomic abnormalities such as mutations, deletions, duplications or amplifications. This cytogenetic technique has the advantage that it can be applied on stored DNA of a patient sample rather than metaphase chromosomes which are difficult to gain from tumor cells in the required amount and quality [16].

The development of array-CGH (aCGH) provided the possibility for a genome-wide analysis with a high resolution [28]. It is based on the concurrent hybridization of DNA targets arrayed on a solid platform such as glass with differentially labeled genomic DNA of test and reference samples with green and red fluorochromes respectively. The advantage over fluorescence in situ hybridization (FISH) is that DNA alterations in a genome can be detected simultaneously at multiple loci, which is useful for diagnostic applications [29]

However, balanced rearrangements or some mosaics cannot be identified by array based CGH and it cannot be used to determine the genomic position of inserted or deleted segments [18]. aCGH offers a high diagnostic yield for the identification of clinically important genomic aberrations and thus can be used as the first-tier diagnostic test for this purpose. However, additional G-banding and/or FISH analyses after abnormal chromosomal microarray analysis (CMA) test may need to be included to ensure accurate risk estimates. Further, after normal CMA, an abbreviated karyotype analysis or other confirmatory testing should be considered as some abnormalities cannot be detected by aCGH [16,30]. Comparative genomic hybridization tests were GTR registered as cytogenetic tests in a combined approach with Next-Generation (NGS)/Massively parallel sequencing to test for TP53 mutations, the RAS and FLT3 gene. CGH was also detected in a single technique approach for further relevant biomarkers of the listed drugs; however, in such cases it was registered as a test of the GTR category “Molecular Genetics” and was therefore not evaluated in these analyses.

Furthermore, in combined and single application Next-Generation (NGS)/Massively parallel sequencing was only registered as a test of the GTR category “Molecular Genetics”. However, NGS, which includes a variety of methods, is increasingly applied for cytogenetic analyses [10,31]. It already supplements conventional cytogenetic approaches and has the potential to replace them in certain applications in the future [32]. The evaluation of the largest GTR category “Molecular Genetics” would have exceeded the scope of this review. The results for this category which also includes NGS and other sequencing based techniques will be evaluated and discussed in a further approach.

### 3.2. Biochemical Genetic Testing Techniques

Biochemical genetic tests to analyze drug response could not be identified via screening of the GTR by drug. However, via a screening by the according biomarker, registered biochemical genetic tests (Table 3) for about 20.6% (Supplementary Material 3) of the listed drugs with required or recommended testing prior to prescription in the EU and one of 32 drugs with annotations on actionable pharmacogenomics in the drug label provided by the respective EPAR were identified (Supplementary Material 2).

#### 3.2.1. Immunohistochemistry

Immunohistochemistry (IHC) is an essential application for the diagnosis of cancers and as companion diagnostic tool in cancer treatment. It requires the availability of tissue samples and can be performed on frozen or formalin-fixed paraffin-embedded (FFPE) tissue. IHC is also suitable for small amounts of tissue acquired in procedures such as core biopsies [38,39].

Thereby, monoclonal or polyclonal antibodies are incubated with the analyzed tissue to determine the distribution of a biomarker antigen of interest. For visualization, mainly enzymes or fluorescent dyes are used which are directly coupled to the primary antibody or to an appropriate secondary antibody but also radioactive elements, or colloidal gold can be applied. The signal is detected dependent on the method of visualization either under an ordinary or fluorescent microscope [38,39].

Diagnostic tests based on immunohistochemistry were identified by GTR screening for biomarkers such as Anaplastic Lymphoma Kinase (ALK), ROS and PD-L1. Although IHC is specified as a method of choice in several EPARs for HER2 overexpression, test for this biomarker were only identified in the GTR test categories “Cytogenetics” (FISH) and “Molecular genetics”.

The biomarker Programmed cell death ligand 1 (PD-L1) is crucial for the selection of patients with e.g., advanced non-small-cell lung cancer (NSCLC) who would respond best to treatment with PD-L1 inhibitors. Unique immunohistochemical assays were developed for each inhibitor. However, several studies analyzing interlaboratory concordance found moderate to high agreement levels for various assays and laboratory developed tests [40]. For detection of ALK gene rearrangements that lead to fusion genes, fluorescence in situ hybridization (FISH) has been used as the gold standard. Nevertheless, for screening and diagnostics, also IHC is commonly used to detect ALK protein expression due to its very high sensitivity and specificity in identifying ALK positive lung cancers in concordance with FISH results [41]. Still, in some cases between FISH and IHC results discrepancy was observed [42]. In tumors such as i.e., breast carcinoma, IHC is commonly used to predict drug response. Thereby, the presence of hormone receptors and/or human epidermal growth factor receptor 2 is analyzed [38,39].

Due to technical artifacts and differences of sensitivity between differing antibodies and pretreatments of tissue, a lack of inter laboratory standardization of the IHC technique analyzing the positivity of HER2 and hormone receptor staining was observed. Furthermore, variability in interpretation by a pathologist occurs subjectively. Therefore, also methodological comparisons of kits that have been introduced (such as HercepTest) were observed with mixed results [22,39]. Confirmatory test results obtained by alternative techniques are therefore crucial and are recommended in several EPARs in case of equivocal test results with IHC.

#### 3.2.2. Enzyme Assays and Mass Spectrometry

Enzyme assays for lysosomal enzymes such as beta-glucocerebrosidase and alpha-galactosidase A are registered in the GTR and can be applied for guided treatment with Velaglucerase alfa in type 1 Gaucher disease (GD) and Migalastat in Fabry disease respectively. For both drugs, testing is required according to the respective EPARs. However, the EPARs of these drugs do not specify any testing technique [43,44].

Enzyme assays are applied to either identify a target enzyme or to determine the quantity of the enzyme in a sample [45,46]. Thereby, enzyme assays measure product accumulation or substrate consumption over time. Many enzymes can be assayed via several different approaches. Furthermore, to measure the concentration of products or substrates, different methods can be used. Besides optical methods (e.g., fluorometric, calorimeteric or a photometric assay), also electrochemical methods are utilized such as pH determinations for reactions involving pH changes [45].

Enzyme assays based on fluorometry are optical techniques measuring the emission of fluorescence on the breakdown of e.g., the most commonly used specific 4-methylumbelliferone synthetic substrate and release of fluorescent 4-MU, which is directly proportional to the activity of the enzyme in for example dried blood samples. The reaction is catalyzed by specific enzymes; therefore, dependent on the enzyme utilized, the assay protocol can vary [47,48]. However, the identification of enzyme activity cannot detect heterozygote GBA carriers of GD or the disease phenotype and in Fabry disease, an X-linked disorder, determination of GLA mutation by enzyme activity in female persons is limited. Enzyme assays can be used as a first-tier test and should be further confirmed by mutation analysis. Still, molecular genetic analysis of the GLA gene is an important diagnostic tool in women [49,50,51,52]. For targeted therapy of lysosomal storage disorders like Fabry disease or Gaucher disease, enzyme assays including fluorometry and tandem mass spectrometry (MS/MS) methods to identify deficient enzyme activity have been the gold standard as diagnostic tests [50,51,53,54,55].

For measurement of enzyme activity, leukocytes, cultured fibroblasts or dried blood spots can be used [54,56]. A comparison of the MS/MS and fluorescence assay indicated that MS/MS provided improved specificity in detection of *GBA* mutation carriers compared to the fluorescence assay. Therefore, results by fluorescence assays require confirmation by genotyping [57]

For analysis by tandem mass spectrometry, for each assay an internal standard is used to determine the enzyme activity. Molecular and atomic masses of enzyme products are analyzed via mass spectrometry by breaking each molecule into ionized fragments. The fragments are separated and analyzed according to their mass-to-charge ratio. MS/MS can also be used to quantify molecular species [50,58]. Coupling of liquid chromatography or gas chromatography to a mass spectrometer allows a more precise identification of substances such as metabolites in a sample [58,59,60].

## 4. Discussion

In the EU, mainly genetic tests are recommended or required for drugs authorized for cancer treatment. Therefore, despite the increasing utilization of molecular genetic techniques like qRT-PCR or next-generation sequencing and combined strategies, the diagnosis of chromosomal disorders by classical cytogenetic methods such as fluorescence in situ hybridization and biochemical genetic techniques such as immunohistochemistry remained a standard for guided therapy with several drugs uniformly approved in the European Union [11].

Cytogenetic analyses provide the possibility to gain information on genetic abnormalities on single cell level [61]. Current techniques with a high resolution can detect DNA deletions or duplications of a few hundred thousand nucleotides [62].

Chromosome staining like Giemsa banding (G-banding) is an early method used since the 1970s for the detection of cytogenetic abnormalities and is still applied in clinical diagnostics. However, also molecular cytogenetic technologies that incorporate nucleic acid-based probes such as fluorescence, silver and chromogenic in situ hybridization (CISH and FISH), comparative genomic hybridization (CGH) and its respective further development to copy-number arrays for chromosomal microarray analysis (CMA), were developed for a higher resolution in the detection of chromosomal and genetic abnormalities. However, the utilization of targeted probes does not allow genome-wide analyses by in situ hybridization, and CMA cannot detect balanced rearrangements or determine the chromosomal position of inserted or deleted segments [16]. Due to this technical limitation, it is crucial to evaluate the relative strengths and weaknesses of each technique to determine the best approach for the respective application and analyzed condition in clinical diagnostics [10]. To verify abnormalities identified by a first-tier method, in general, targeted analysis may be suitable.

HER2 alterations like HER2 protein overexpression, HER2 gene amplification and HER2 gene mutations can be identified and assessed by several laboratory methods in Non-small cell lung cancer (NSCLC). Immunohistochemistry (IHC) is applied to test for HER2 positivity due to protein overexpression and genetic alterations are identified by next generation sequencing (NGS) and fluorescent in situ hybridization (FISH). However, a gold standard has not been established yet [63].

GTR entries for tests including combinations of several techniques were often misleading as information on whether the relevant biomarker was targeted by all techniques was inconclusive. Therefore, additional information on the applied methodology on the provider websites is crucial for a first assessment of the respective tests. Due to such information, it was identified that for none of the biomarkers relevant for the listed drugs of the “Actionble PGx” fraction techniques of the category “Cytogenetics” were actually applied, although the according technique entries in the GTR are presented for several of these markers; however, techniques registered in a combination could not be assigned to a specific marker in the multigene approaches. Therefore, while GTR also presented several entries, for only one biomarker of the category “Biochemical Genetics”, a biochemical genetic technique was actually applied according to the provider websites. Here, it furthermore was a combined approach with a molecular genetic technique, as mainly these biomarkers (“Actionble PGx”) were analyzed by techniques categorized as “Molecular Genetics”. Thereby an emphasis on NGS was detected. Still, some websites, to which the user is directed by the Universal Resource Locator (URL) provided by the GTR, do not offer sufficient transparency on the applied techniques.

A tendency of multigene testing using panels for certain disease conditions and combined approaches of several techniques was observed in a majority of offered test services. A clinical laboratory geneticist should therefore assess which method or combination of methods is appropriate also in accordance with the referral reason [10]. Thereby, it is a requirement to ensure the utilization of validated tests as indicated in the SmPCs of the respective EPAR recommending or demanding a diagnostic test prior to drug prescription.

Although EMA mainly does not provide guidance for the choice of companion diagnostic tests or techniques, according to drug label information provided by the respective EPAR, eligibility for treatment with Lapatinib, Trastuzumab and Trastuzumab emtansin is only warranted if HER2 overexpression status is determined by an IHC 3+ score, which can be achieved by applying immunohistochemistry to test the affected patient tissue [64,65,66]. Currently, immunohistochemistry (IHC) and fluorescence in situ hybridization (FISH) are regarded as standard methods for the determination of HER2 status in breast cancer, and some of them have been approved by the U.S. Food and Drug Administration (FDA) for HER2 testing in breast cancer since 1998 [13]. However, a screening of test methods by drug and biomarker showed that IHC testing regarding drug response is not registered as an offered testing method in the GTR database although the test method category “Biochemical Genetics” is provided. GTR presented tests and services offered worldwide are registered on an optional basis. Therefore, several biochemical genetic and cytogenetic methods applied for diagnostic tests in clinical practice such as CISH and SISH as cheaper and easier alternatives to FISH were not registered as an offered test method in the GTR database. In addition, sufficient evidence on test validity and utility is rarely included. Therefore, a comprehensive registry that covers all tests, testing techniques and information on test validity for the according drug responses and biomarkers relevant for drug prescriptions is not yet available.

According to current literature, there are emerging technologies that have the potential for pharmacogenetic testing in clinical use and therefore may be commercially available in future. These are molecular genetic techniques such as custom-target sequencing and long-read sequencing approaches [67,68]. Thus, further evaluations of the GTR test category “Molecular Genetics” will elucidate current commercially available molecular genetic techniques and emerging techniques with a potential for future clinical applications.

## 5. Conclusions

Information on suitable techniques for predictive biomarker testing is scarcely provided in the SmPCs in Europe, also with regard to new drugs for targeted therapy in oncology. Furthermore, developments concerning predictive biomarkers and testing techniques are quite fast, which provides a challenge. Therefore, a focus on standardization and validation of pharmacogenetic tests will be highly essential. Especially, since the importance of genetic tests as companion diagnostics is increasing with the available and harmonized information on pharmacogenetic and/or pharmacogenomic implications provided in drug labels. The demand for genetic testing guided treatment to increase the safety of drug therapy therefore could grow and may be accelerated by patients’ concern to receive optimal treatment. Therefore, prescribers will need facilitated access to reliable information on availability, validity and utility of genetic and genomic testing. Currently a comprehensive registry that meets the emerging needs for information on genetic tests that could be applied as companion diagnostics in the EU is not available.

## Figures and Tables

**Table 1:** GTR-registered tests identified via screening by drug. IHC: Immunohistochemistry, FISH: Fluorescence in situ hybridization, SISH: Silver in situ hybridization. According to entries of the Genetic Testing Registry for EMA approved drugs, for which testing is recommended or mandatory or annotations on actionable pharmacogenomics are present (according to the respective EPAR).

Drug	Disease	Gene/Biomarker	Eligibility for Treatment According to EPAR	Commercially Offered Cytogenetic Test *
Crizotinib	Lung cancer	ALK or ROS1	ALK-positive or ROS1-positive NSCLC status EPAR: Xalkori 2020	Fluorescence In Situ Hybridization, Test combination (Cytogenetic technique + Molecular genetic technique): RT-PCR with gel analysis (Chromosomal breakage study) + Allele-specific primer extension (ASPE) (Applied Biosystems^TM^ 7900HT Sequence Detection System)
Lapatinib	Breast cancer	ERBB2	HER2 (ErbB2) overexpressed in tumours defined by IHC3+, or IHC2+ with gene amplification or gene amplification alone EPAR: Tyverb 2019	Fluorescence In Situ Hybridization
Trastuzumab	Breast and gastric cancer	ERBB2	Breast cancer: “HER2 overexpression or HER2 gene amplification as determined by an accurate and validated assay.” Gastric cancer: “IHC2+ and a confirmatory SISH or FISH result, or by an IHC 3+ result” EPAR: Herceptin 2020	Fluorescence In Situ Hybridization
Trastuzumab emtansin	Breast cancer	ERBB2	“HER2 positive tumour status, defined as a score of 3 + by immunohistochemistry (IHC) or a ratio of ≥ 2.0 by in situ hybridization (ISH) or by fluorescence in situ hybridization (FISH)” EPAR: Kadcyla 2020	Fluorescence In Situ Hybridization

* According to entries of the Genetic Testing Registry for EMA approved drugs, for which testing is recommended or mandatory or annotations on actionable pharmacogenomics are present (according to the respective EPAR).

**Table 2:** Strengths and limitations of GTR-registered cytogenetic tests identified via screening by biomarker for PharmGKB listed drugs with a requirement or recommendation for testing in the EU. CGH: comparative genomic hybridization.

Cytogenetic Method (%)	Strengths	Limitations	Resolution/Biomarker	Literature
G-banding/Karyotyping (10.3%)	Highly informative on chromosome level	Low resolution	~5–10 Mb/Biomarker: e.g., Philadelphia-Chromosome, t(15;17) translocation, PML/RAR-alpha	Silva et al. (2019) [10] Rack et al. (2019) [11]
FISH (51.7%)	High specificity, reliable localization and targeted analysis possible	No genome-wide analysis possible, specific probes required	~100 kb/Biomarker: e.g., ALK, HER2, Philadelphia-Chromosome	Silva et al. (2019) [10] Rack et al. (2019) [11]
CGH (combined with NGS) (5.2%)	Genome-wide analysis possible	No detection of balanced rearrangements, mosaicism, inversions	~5–10 Mb/Biomarker: e.g., Philadelphia-Chromosome, t(15;17) translocation, PML/RAR-alpha	Silva et al. (2019) [10] Weise et al. (2019) [12]

**Table 3:** Strengths and Limitations of biochemical genetic testing methods registered in the GTR for the PharmGKB-listed drugs with required or recommended testing according to the respective EPARs.

Biochemical Genetic Testing Method (%)	Strengths	Limitations	Literature
Immunohistochemistry (10.3%)	Easy to perform	Operator variability	Taylor (2015), Matos et al. (2010) [33,34]
Enzyme assay/Fluorometry (10.3%)	High sensitivity, measurement of concentration possible	Relies on highly purified protein samples	Sorenson et al. (2020) [35]
Metabolyte analysis/Gas chromatography–mass spectrometry (3.4%)	Less costly and time-consuming	Derivatization artefacts	Mattison et al. (2004), Aretz and Meierhofer (2016) [36,37]

## Data Availability

Data supporting reported results are provided in Supplementary material 1–3. Results were obtained in 2020 on the basis of PharmGKB data (https://www.pharmgkb.org/labelAnnotations accessed on 15 June 2021) and data provided by NCBI Genetic Testing Registry (https://www.ncbi.nlm.nih.gov/gtr/ accessed on 15 June 2021) for the respective drugs and biomarkers.

## Supplementary Materials

The following are available online at https://www.mdpi.com/article/10.3390/diagnostics11071169/s1, Supplementary Material 1: GTR-registered tests of the category “Cytogenetics” screened by biomarker (testing required or recommended prior to prescription of the respective drug), Supplementary Material 2: GTR-registered tests of the category “Cytogenetics” and “Biochemical Genetics” by biomarker (drugs with annotations on actionable pharmacogenomics in the drug label provided by the respective EPAR), Supplementary Material 3: GTR-registered tests of the category “Biochemical Genetics” screened by biomarker (testing required or recommended prior to prescription of the respective drug).
