# Supplementary material for: Cytogenetic and Biochemical Genetic Techniques for Personalized Drug Therapy in Europe
Source: Diagnostics (Basel). 2021 Jun 26;11(7):1169. doi: 10.3390/diagnostics11071169 (PMC8303692; doi:10.3390/diagnostics11071169)
Supplement: Supplementary file 1 [file diagnostics-11-01169-s001.zip › diagnostics-1249381-supplementary.pdf]

diagnostics

MDPI

Supplementary Material

Cytogenetic and Biochemical Genetic Techniques for Personalized Drug Therapy in Europe

Tatjana Huebner *, Catharina Scholl and Michael Steffens

[1] Research Division, Federal Institute for Drugs and Medical Devices, 53175 Bonn, North Rhine-Westphalia, Germany; Catharina.Scholl@bfarm-research.de (C.S.); Michael.Steffens@bfarm-research.de (M.S.)
* Correspondence: Tatjana.Huebner@bfarm-research.de

Supplementary Material 1. GTR-registered tests of the category "Cytogenetics" screened by biomarker (testing required or recommended prior to prescription of the respective drug)

Drug	Disease	Gene/Biomarker	Cytogenetics
**Abacavir**	HIV infection	HLA-B*5701	-
**Abemaciclib**	Breast cancer	Hormone receptor positive (PGR, ESR1, ESR2), HER2 negative (ERBB2)	ERBB2: Fluorescence in situ hybridization
**Afatinib**	Lung cancer	EGFR	Fluorescence in situ hybridization
**Alectinib**	Lung cancer	Anaplastic Lymphoma Kinase (ALK)	Fluorescence In Situ Hybridization, RT-PCR with gel analysis (Cytogenetics)+ Molecular genetics: Allele-specific primer extension (ASPE) (Applied Biosystems™ 7900HT Sequence Detection System) (Molecular genetics), Fluorescence In Situ Hybridization Molecular genetics: Next-Generation (NGS)/Massively parallel sequencing (MPS)
**Arsenic trioxide**	Acute promyelocytic leukaemia	t(15;17) translocation, PML/RAR-alpha (PML (15q24.1), RARA (17q21.2)	t(15;17) translocation: G-banding PML/RAR-alpha: Fluorescence in situ hybridization (FISH)
**Atazanavir**	HIV infection	CYP2C19 *2,*3 or other star alleles related to decreased enzyme function	-
**Atezolizumab**	Urothelial cancer, lung cancer, triple-negative breast cancer	PD-L1	Fluorescence in situ hybridization (FISH)
**Binimetinib**	Melanoma	BRAF-V600 Mutation	-
**Bosutinib**	Chronic myelogenous leukaemia	Philadelphia-Chromosome	Fluorescence in situ hybridization (FISH), FISH (M)+G-banding, G-banding
**Brentuximab vedotin**	Hodgkin lymphoma and anaplastic large cell lymphoma	TNFRSF8	-
**Brigatinib**	Lung cancer	ALK	Fluorescence In Situ Hybridization, RT-PCR with gel analysis (Cytogenetics)+ Molecular genetics:

			Allele-specific primer extension (ASPE) (Applied Biosystems 7900HT Sequence Detection System), Fluorescence In Situ Hybridization+Molecular genetics: Next-Generation (NGS)/Massively parallel sequencing (MPS)
**Capecitabine**	Colorectal Neoplasms	DPYD (including DPYD*2A, c.1679T>G, c.2846A>T and c.1236G>A/HapB3 variants)	-
**Carglumic acid**	Hyperammonaemia	NAGS	-
**Ceritinib**	Lung cancer	Anaplastic Lymphoma Kinase	Fluorescence In Situ Hybridization, RT-PCR with gel analysis (Cytogenetics)+ Allele-specific primer extension (ASPE) (Applied Biosystems 7900™HT Sequence Detection System) (Molecular genetics), Fluorescence In Situ Hybridization ((Cytogenetics)+Next-Generation (NGS)/Massively parallel sequencing (MPS) (Molecular genetics)
**Cerliponase alfa**	Neuronal ceroid lipofuscinosis type 2 (CLN2) disease	TPP1	-
**Cetuximab**	Gastric cancer	Kras, Nras	Comparative Genomic Hybridization+ Molecular Genetics: Next-Generation (NGS)/Massively parallel sequencing (MPS)
**Cholic acid**	Inborn errors in primary bile acid synthesis due to Sterol 27-hydroxylase (presenting as cerebrotendinous xanthomatosis, CTX) deficiency, 2- (or α-) methylacyl-CoA racemase (AMACR) deficiency or Cholesterol 7α-hydroxylase (CYP7A1) deficiency in infants, children and adolescents aged 1 month to 18 years and adults	CYP27A1, AMACR and CYP7A1, HSD3B7,AKR1D1	-
**Cobimetinib**	Melanoma	BRAF V600	-
**Crizotinib**	Lung cancer	ALK, ROS	Fluorescence In Situ Hybridization, RT-PCR with gel analysis (Cytogenetics) + Molecular Genetics: Allele-specific primer extension (ASPE)

			(Applied Biosystems™ 7900HT Sequence Detection System), Fluorescence In Situ Hybridization + Molecular Genetics: Next-Generation (NGS)/Massively parallel sequencing (MPS)
**Dabrafenib**	Melanoma	BRAF V600	-
**Dacomitinib**	Lung cancer	EGFR (ErbB-1)	Fluorescence in situ hybridization (FISH)
**Dasatinib**	Acute lymphatic leukemia	Philadelphia-chromosome	Fluorescence in situ hybridization (FISH)(M,I), FISH (M)+G-banding, G-banding
**Eliglustat**	Gaucher's disease	CYP2D6	-
**Elosulfase alfa**	Mucopolysaccharidosis, type IVA (Morquio A Syndrome, MPS IVA)	GALNS	-
**Encorafenib**	Unresectable or metastatic melanoma	BRAF V600	-
**Erlotinib**	Lung cancer	EGFR (ErbB-1)	Fluorescence in situ hybridization (FISH) (F,I),
**Everolimus**	Breast cancer	ERBB2	Fluorescence In Situ Hybridization (F, T)
**Fulvestrant**	Breast cancer	PGR gene residing on chromosome 11q22, ESR1 (6q25.1), ESR2 (14q23.2)	-
**Gefitinib**	Lung cancer	EGFR (ErbB-1)	Fluorescence in situ hybridization (FISH) (F,I),
**Ibrutinib**	Chronic lymphocytic leukemia	17p deletion or TP53 mutation	17p deletion:<br><br>Digital / Virtual karyotyping (M)+G-banding+ Molecular genetics: Microarray, G-banding, Fluorescence in situ hybridization +Microarray (e.g., Affymetrix® Cytoscan® HD Array)<br><br>TP53:<br><br>Comparative Genomic Hybridization + Molecular Genetics: Next-Generation (NGS)/Massively parallel sequencing (MPS), Fluorescence in situ hybridization (FISH)(F)+G-banding, Fluorescence in situ hybridization (FISH) (I,M,F)<br>(e.g., Affymetrix® GeneChip® Scanner 3000 7G Whole-Genome Association System)
**Imatinib**	Acute lymphatic and chronic myeloid leukemia	Philadelphia-chromosome	Fluorescence in situ hybridization (FISH)(M,I), FISH (M)+G-banding, G-banding
**Ivacaftor**	Mucoviscidosis	CFTR mutations	G-banding+ Molecular Genetics:

			Allele-specific PCR+PCR with allele specific hybridization, G-banding+ Molecular Genetics: PCR+Next-Generation (NGS)/Massively parallel sequencing (MPS)+ Bi-directional Sanger Sequence Analysis
**Lapatinib**	Breast cancer	HER2 overexpression, ERBB2	Fluorescence In Situ Hybridization
**Lomitapid**	Increased cholesterol or fat levels	LDLR, APOB and PCSK9	-
**Lorlatinib**	Non-small cell lung cancer	ALK	Fluorescence In Situ Hybridization (T,I), RT-PCR with gel analysis (Cytogenetics)+ Molecular Genetics: Allele-specific primer extension (ASPE) (Applied Biosystems™ 7900HT Sequence Detection System), Fluorescence In Situ Hybridization + Molecular Genetics: Next-Generation (NGS)/Massively parallel sequencing (MPS)
**Lumacaftor / Ivacaftor**	Cystic fibrosis	F508del mutation in the CFTR gene	-
**Midostaurin**	Acute myeloid leukaemia	FLT3	Comparative Genomic Hybridization)+ Molecular Genetics: Next-Generation (NGS)/Massively parallel sequencing (MPS)
**Migalastat**	Fabry disease	GLA	-
**Neratinib**	Breast cancer	HER2/ERBB2	Fluorescence In Situ Hybridization
**Nilotinib**	Chronic myeloid leukaemia	Philadelphia-chromosome	Fluorescence in situ hybridization (FISH)(M,I), FISH (M)+G-banding, G-banding
**Olaparib**	Ovarian Cancer	BRCA1/2	-
**Osimertinib**	Non-small cell lung cancer (NSCLC)	EGFR (ErbB-1) T790M	Fluorescence in situ hybridization (FISH)
**Palbociclib**	HR-positive, HER2-negative locally advanced or metastatic breast cancer	PGR, ESR1, ESR2, ERBB2	ERBB2: Fluorescent in situ hybridization
**Panitumumab**	Colon Cancer	RAS	-
**Pembrolizumab**	Non-small cell lung carcinoma (NSCLC) Head and neck squamous cell carcinoma (HNSCC) Urothelial carcinoma	PD-L1	Fluorescence in situ hybridization (FISH) (F,T)
**Pertuzumab**	Breast cancer	ERBB2	Fluorescent in situ hybridization (I, T, F)
**Ponatinib**	Chronic myeloid leukaemia (CML)	Philadelphia chromosome	Fluorescence in situ hybridization (FISH)(M,I), FISH (M)+G-banding, G-banding
**Ribociclib**	Breast cancer,	PGR, ESR1, ESR2, ERBB2	ERBB2: Fluorescent in situ

	HR-positive, HER2-negative		hybridization (I,F)
**Rituximab**	CD20-positive diffuse large V cell non-Hodgkin's lymphoma	MS4A1	-
**Rucaparib**	Relapsed or progressive epithelial ovarian cancer (EOC), fallopian tube cancer (FTC), or primary peritoneal cancer (PPC)	BRCA	-
**Talazoparib**	Breast cancer	BRCA1/2, HER2	ERBB2: Fluorescent in situ hybridization
**Tezacaftor / Ivacaftor**	Cystic fibrosis	F508del, CTFR	-
**Trametinib**	Melanoma, Non-small cell lung cancer (NSCLC)	BRAF V600	-
**Trastuzumab**	Breast and gastric cancer	HER2/ERBB2	Fluorescence In Situ Hybridization (I,F)
**Trastuzumab emtansin**	Breast cancer	HER2/ERBB2	Fluorescence In Situ Hybridization (I,F)
**Vandetanib**	Medullary thyroid cancer (MTC)	RET	Fluorescence In Situ Hybridization, Fluorescence in situ hybridization (FISH)+ Molecular Genetics: Next-Generation (NGS)/Massively parallel sequencing (MPS)+ Biochemical Genetics: Protein Expression (PD-L1) (e.g., NeoTYPE® Discovery Profile for Solid Tumors)
**Velaglucerase alfa**	Type 1 Gaucher disease	GBA	-
**Vemurafenib**	Melanoma	BRAF V600	-

M: Metaphase, I: Interphase, F: Fluorescence In Situ Hybridization, T: Targeted variant analysis.

Supplementary Material 2. GTR-registered tests of the category "Cytogenetics" and "Biochemical Genetics" by biomarker (drugs with annotations on actionable pharmacogenomics in the drug label provided by the respective EPAR).

Drug	Disease	Gene/Biomarker	Cytogenetics	Biochemical Genetics
**Amifampridine Phosphate**	Lambert–Eaton myasthenic syndrome	NAT2	-	-
**Aripiprazole**	Schizophrenia	CYP2D6	-	-
**Brexpiprazole**	Schizophrenia	CYP2D6	-	-
**Brivaracetam**	Epilepsy	CYP2C19 (poor metabolism)	-	-
**Clopidogrel**	Secondary prevention of atherothrombotic events, Prevention of atherothrombotic and thromboembolic events in atrial fibrillation	CYP2C19 intermediate or poor metabolism	-	-
**Darifenacin (hydrobromide)**	Urge incontinence and/or increased urinary frequency and urgency as may occur in adult patients with overactive bladder syndrome	CYP2D6 (poor metabolism)	-	-
**Dolutegravir**	HIV infection	UGT1A1 (poor dolutegravir metabolism)	-	-
**Duloxetine**	Treatment of major depressive disorder. Treatment of diabetic peripheral neuropathic pain. Treatment of generalised anxiety disorder.	CYP2D6	-	-
**Efavirenz**	HIV infection	Homozygous G516T genetic variant of the CYP2B6 isoenzyme	-	-
**Efavirenz / Emtricitabine / Eenofovir Disoproxil**	HIV infection	Homozygous G516T genetic variant of the CYP2B6 isoenzyme	-	-
**Eltrombopag**	Chronic immune (idiopathic) thrombocytopenic purpura (ITP)	Factor V Leiden (F5 gene) and ATIII deficiency (SERPINC1 gene)	-	F5: Electromagnetic Clot Detection/Quantitative Enzymatic (Analyte)+ Molecular Genetics: Polymerase Chain Reaction/Fluorescence Monitoring, Clotting (A)+ Molecular Genetics: Polymerase Chain Reaction/Fluorescence Monitoring
**Erlotinib**	Non-small-cell lung cancer (NSCLC)	UGT1A1	-	-

**Ethinyl estradiol / norelgestromin**	Female contraception	Predisposition for venous thromboembolism (including F5), antithrombin-III deficiency (SERPINC1), protein C deficiency (PROC) and protein S deficiency (PROS))	-	-
**Fesoterodine**	Treatment of the symptoms that may occur with overactive bladder syndrome.	CYP2D6 (poor metabolisers)	-	-
**Gefitinib**	Advanced or metastatic non-small cell lung cancer	CYP2D6 (poor metabolism)	-	-
**Glibenclamide**	Neonatal diabetes mellitus	G6PD (deficiency)	-	-
**Glimepiride**	Type 2 diabetes mellitus	G6PD (deficiency)	-	-
**Irinotecan**	Metastatic adenocarcinoma of the pancreas	Homozygozity for UGT1A1*28 allele	-	-
**Lapatinib**	Breast cancer, whose tumours overexpress HER2 (ErbB2)	HLA-DQA1*02:01 and HLA-DRB1*07:01	-	-
**Lesinurad**	Hyperuricaemia in gout patients	CYP2C9 (poor metabolism)	-	-
**Lidocaine / Prilocaine**	Primary premature ejaculation	G6PD	-	-
**Mercaptopurine**	Acute lymphoblastic leukaemia (ALL)	TPMT, NUDT15	-	-
**Methylene blue**	Medicinal and chemical products-induced methaemoglobinaemia	G6PD, NADPH deficiency	-	-
**Pazopanib**	Advanced renal cell carcinoma	HLA-B	-	-
**Pioglitazone**	Type 2 diabetes mellitus	G6PD	-	-
**Pioglitazone**	Stable angina pectoris	CYP2D6	-	-
**Ranolazine**	Stable angina pectoris	CYP2D6 (poor metabolism)	-	-
**Rasburicase**	Acute hyperuricaemia	G6PD	-	-
**Regorafenib**	Metastatic colorectal cancer	KRAS	-	-
**Ticagrelor**	Prevention of atherothrombotic events	CYP2C19 (loss of function allele)	-	-
**Toremifen**	Hormone-dependent metastatic breast cancer	ESR1 and ESR2	-	-
**Vortioxetine**	Major depressive episodes	CYP2D6 (poor metabolism, ultra-rapid metabolism)	-	-

Supplementary Material 3. GTR-registered tests of the category "Biochemical Genetics" screened by biomarker (testing required or recommended prior to prescription of the respective drug)

Drug	Disease	Gene/Biomarker	Biochemical Genetics
**Alectinib**	Lung cancer	ALK	Immunohistochemistry for ALK and ROS fusion proteins(I)+ Molecular Genetics: Pyrosequencing
**Atezolizumab**	Urothelial cancer, lung cancer, triple-negative breast cancer	PD-L1	Immunohistochemistry
**Brigatinib**	Lung cancer	ALK	Immunohistochemistry for ALK and ROS fusion proteins(I)+ Molecular Genetics: Pyrosequencing
**Capecitabine**	Colorectal Neoplasms	DPYD (including DPYD*2A, c.1679T>G, c.2846A>T and c.1236G>A/HapB3 variants)	Gas chromatography–mass spectrometry (GC-MS) (Analyte analysis) (A), Metabolite analysis (A) + Enzyme diagnostics(E) + Molecular Genetics: Bi-directional Sanger Sequence Analysis
**Carglumic acid**	Hyperammonaemia	NAGS	Metabolite levels (A) + Bi-directional Sanger Sequence Analysis(C) (Molecular Genetics),<br>Enzymatic levels (E)+ Molecular Genetics: Multiplex Ligation-dependent Probe Amplification (MLPA) + Trinucleotide repeat by PCR or Southern Blot +Bi-directional Sanger Sequence Analysis+ Next-Generation (NGS)/Massively parallel sequencing (MPS) (C)
**Ceritinib**	Lung cancer	Anaplastic Lymphoma Kinase	Immunohistochemistry for ALK and ROS fusion proteins(I)+Pyrosequencing
**Cerliponase alfa**	Neuronal ceroid lipofuscinosis type 2 (CLN2) disease	TPP1	Enzyme activity(E)+ Molecular Genetics: Bi-directional Sanger Sequence Analysis(C)<br>Fluorometry (E)
**Ivacaftor**	Mucoviscidosis (cystic fibrosis)	CFTR mutations	Enzyme activity+Molecular genetics: PCR with allele specific hybridization, Enzymatic levels+Molecular genetics: Next-Generation (NGS)/Massively parallel sequencing (MPS),Enzymatic levels+Molecular genetics: Next-Generation (NGS)/Massively parallel sequencing (MPS)+Trinucleotide repeat by PCR or Southern Blot
**Lorlatinib**	Non-small cell lung cancer	ALK	Immunohistochemistry for ALK and ROS fusion proteins(I) + Molecular Genetics: Pyrosequencing
**Migalastat**	Fabry disease	GLA	Enzyme activity (E),<br>Enzyme activity (E) + Molecular Genetics: Bi-directional Sanger Sequence Analysis (e.g., Applied Biosystems™ 3500xL capillary sequencing instrument)
**Pembrolizumab**	Non-small cell lung carcinoma (NSCLC)<br><br>Head and neck squamous	PD-L1	Immunohistochemistry (I),<br>Protein expression (I)<br>Antibodies, conjugates, and chromogens (I)

	cell carcinoma (HNSCC)<br>Urothelial carcinoma		
**Velaglucerase alfa**	Type 1 Gaucher disease	GBA	Enzyme activity<br>Fluorometry (Enzymes:beta-Glucosidase) (E)+ Molecular Genetics: Bi-directional Sanger Sequence Analysis +PCR / RFLP,<br>Enzymatic levels (E),<br>Fluorometry (E)+Bi-directional Sanger Sequence Analysis+PCR / RFLP (Molecular Genetics)<br>Enzyme activity (E)+Bi-directional Sanger Sequence Analysis (Molecular Genetics)

I: Immunohistochemistry, A: Analyte, E: Enzyme assay, A: Analyte analysis, C: Sequence analysis of the entire coding region.
